# Pilar Cysts of the Head and Neck: A Case Report

**DOI:** 10.7759/cureus.23932

**Published:** 2022-04-07

**Authors:** Rio Varghese, Fabrice Yabit, Abdulrahman Alrifai, Alexander Burns, Benoit Boucher, Frederick Tiesenga

**Affiliations:** 1 Surgery, Saint James School of Medicine, Park Ridge, USA; 2 Medical School, Avalon University School of Medicine/University of Illinois Chicago, Chicago, USA; 3 Surgery, Saint George’s University, True Blue, GRD; 4 General Surgery, West Suburban Medical Center, Oak Park, USA

**Keywords:** cosmetic dermatologic surgery, risk of rupture, cosmetic surgeirs of facial region, dermal cysts, female patient, multiple surgeries, multiple cysts, surgical intervention, sebaceous cysts, pilar cysts

## Abstract

Pilar cysts, a subtype of sebaceous cysts, are benign masses often commonly found on the scalp, back, and face. They are common amongst women and carry a low potential for malignancy. These lesions arise due to the buildup of keratin in the skin pore, blocking the exit port of sebaceous gland secretions. The buildup of keratin material ultimately leads to cyst formation. The complications of these cysts include inflammation, rupture, infection, and transformation to cancerous lesions in some rare instances. This is a case of a 59-year-old female with a long-standing history of recurrent complicated pilar cysts who presented to the outpatient surgical clinic for assessment of cysts for removal. A total of eleven cysts were successfully removed through two separate surgeries.

## Introduction

Pilar cysts are intradermal cysts derived from the root sheath of the hair follicle, located in the epithelium between the sebaceous gland and the arrector pili muscle. They are lined by stratified squamous epithelium lacking a granular cell layer and filled with keratin and breakdown products. Pilar cysts are firm, slow-growing dermal cysts, and they are the most common skin cysts, which occur when a hair follicle gets blocked by keratin and old shedding skin cells [1]. “Trichilemmal cysts” is another familiar term used to describe pilar cysts. These cysts are part of the sebaceous cysts family and occur in about 10% of the population. Pilar cysts can be found anywhere on the body but are most commonly located on the scalp. These cysts are benign and very rarely become malignant. The tumor form of pilar cysts is known as proliferating trichilemmal cysts, which occur in 3% of cases [2]. These cysts grow slowly and remain benign but can cause discomfort when they ulcerate and become locally aggressive. Pilar cysts should be suspected in any lesion draining yellowish fluid. The diagnosis is made clinically by the presence of a soft, nonpainful mass that grows slowly over time or by imaging when clinical suspicion for other diagnoses is high. The definitive treatment is surgery. The myriad of these cysts in similar locations makes this presentation particularly unique.

## Case presentation

A 59-year-old female patient with a lifetime history of pilar cysts presented to the outpatient clinic with cyst formation predominantly in her head and jaw and with a desire for surgical intervention. She had severe discomfort due to the location of the current cysts. Approximately 11 cysts had appeared over the past 10 years, and all of them were progressively enlarging. The patient recalls that as a child, she had similar cysts that grew on her ear, which were removed surgically when she was in her 20s. She also had two similar masses in her lower back, one of which had ruptured, requiring emergency surgery when both cysts resected post rupture.

Surgical and medical intervention was not sought for the current cysts until recently due to the potential financial burden of not having health insurance. Racial history is notable for German, Irish, Dutch, and Polish. Family history is notable for a brother who had a similar singular cyst. There is no other notable medical history. The patient was not taking any other medications aside from over-the-counter painkillers and multivitamins. Apart from the two surgeries for cysts removal, surgical history is notable for an appendectomy in 2017.

**Figure 1:**
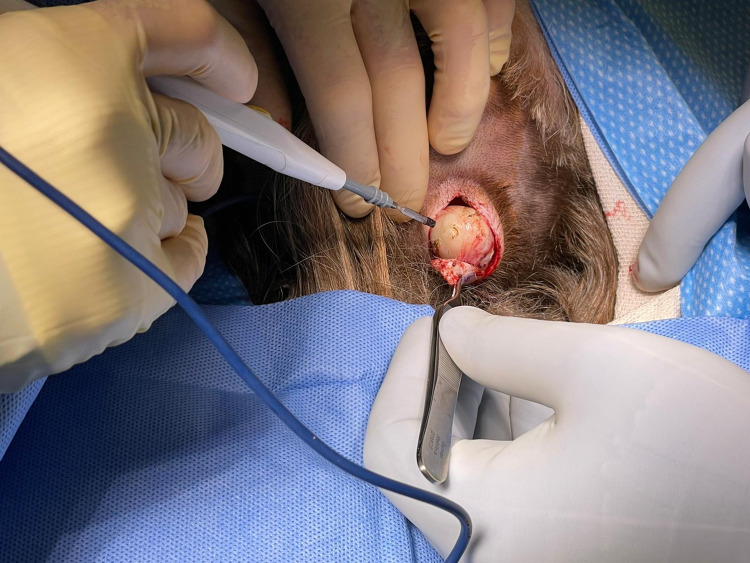
Removal of a cyst from the scalp under general anesthesia in the first surgery.

**Figure 2:**
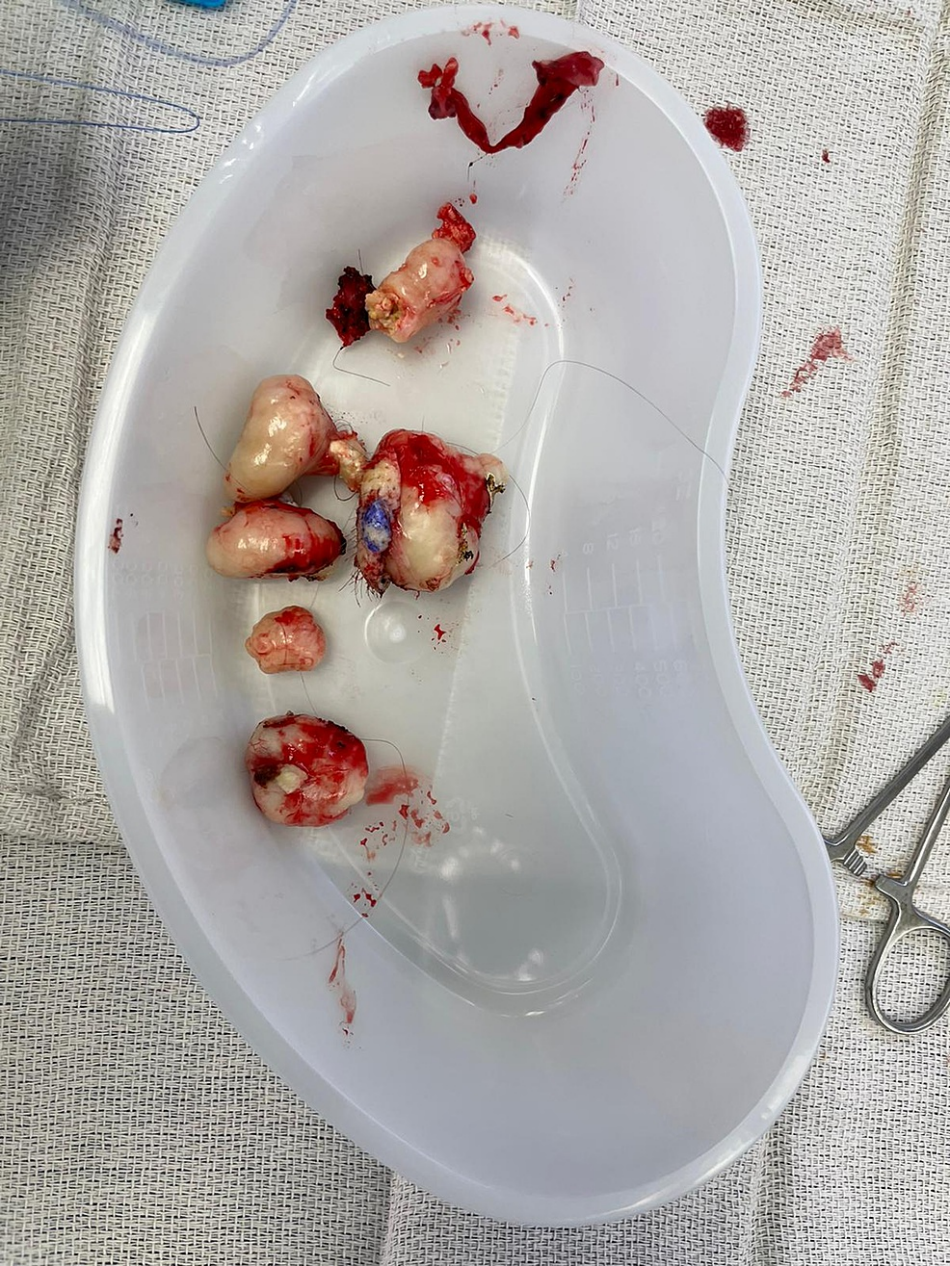
Six separate cysts were extracted during the first surgery.

This report logs two separate surgeries that removed an aggregate of eleven cysts from the patient; the first removed six cysts from the scalp and the second removed four cysts from her scalp and one from her right jaw. A total of six cysts were extracted from the scalp during the first surgery (Figure 1, Figure 2), ranging from 2 x 2 cm to 4 x 4 cm. The wounds were closed after removing these cysts, and meticulous hemostasis was assured.

**Figure 3:**
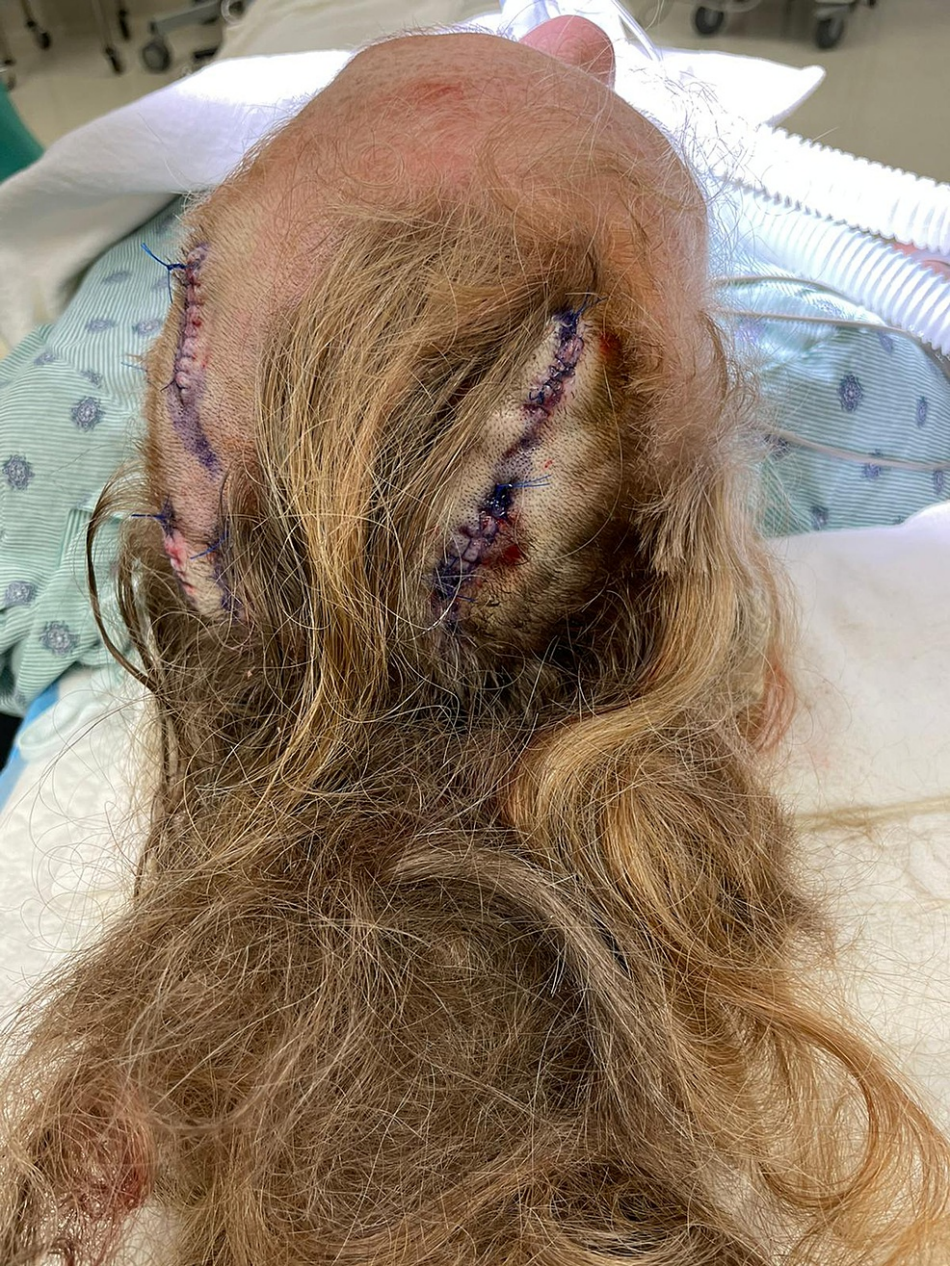
Post-surgery photograph after extraction of cysts from the scalp.

**Figure 4:**
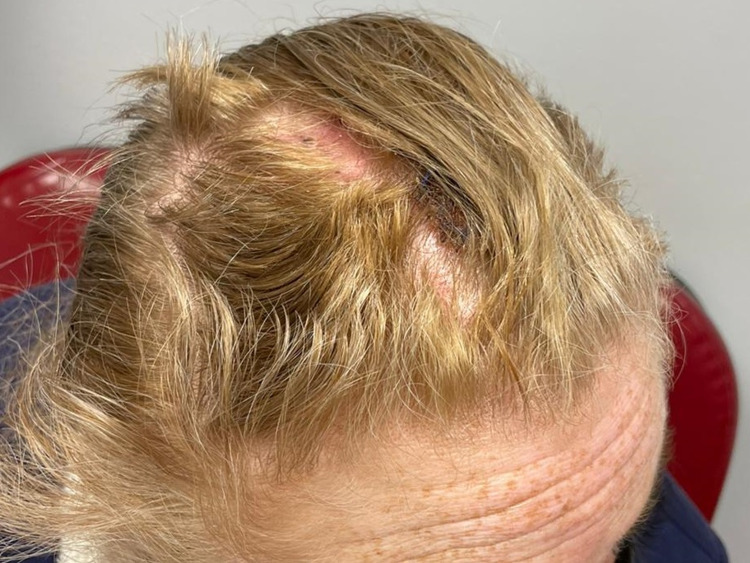
Postoperative check-up, approximately two months after the final surgery.

Approximately 30 days later, the patient underwent a second surgery to remove an additional five cysts (four from other parts of the scalp and one from her right jaw) ranging from 1.1 x 0.5 cm to 2.8 x 2.3cm. The surgical sites were sutured without complications (Figure 3). At the two-month follow-up after her second surgery, the incisions were healing well. Her hair grew back, covering the incision scars (Figure 4).

Pathology reports state that the cysts ranged from 0.8 to 3.8 cm. All the cysts removed in the first surgery were benign pilar cysts (Figure 6). Incidentally, one of the four removed cysts was an epidermal inclusion cyst, while the rest were pilar cysts (Figure 7). 

**Figure 5:**
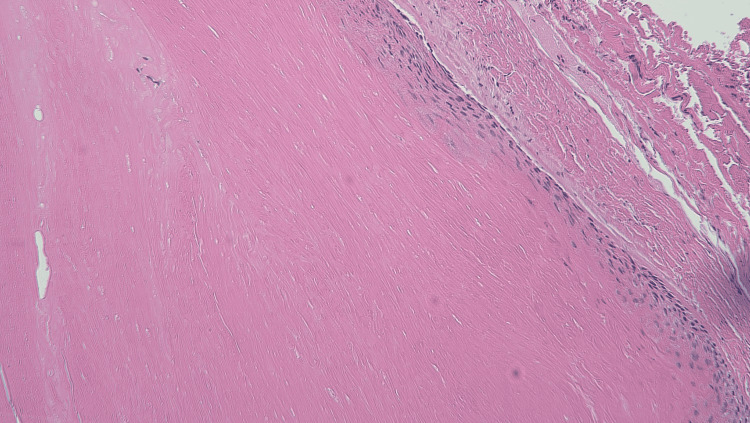
Histological overview of one of the pilar cysts extracted from this patient. The swollen keratin layer is pronounced.

**Figure 6:**
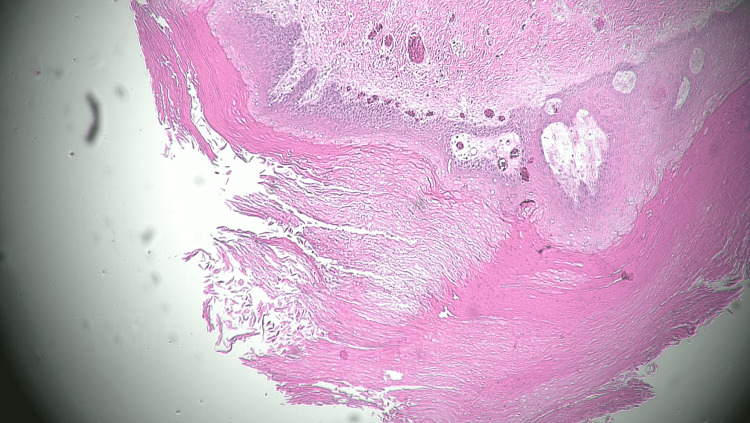
Histological overview of one of the pilar cysts extracted from this patient. The swollen keratinocytes, basal layer, and fibrous capsule are evident.

**Figure 7:**
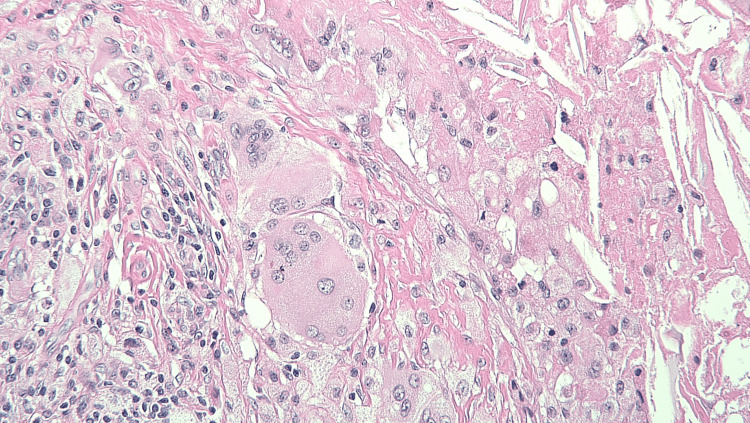
One of the cysts removed was an epidermal inclusion cyst. The key feature is the cyst lining and the lining by stratified squamous epithelium with a granular layer. There is no nuclear atypia, and it contains keratin with a lamellar appearance.

## Discussion

Sebaceous cysts can form anywhere in the body and typically present as asymptomatic, skin-colored dermal nodules, often with a clinically visible central punctum. The lesions can vary from several millimeters to a few centimeters in diameter.

Epidermoid cysts, also called epidermal cysts or epidermal inclusion cysts, are the most common subtype of sebaceous cysts. They can form secondary to trauma, comedo, or follicular epithelium implantation in the dermis. Lesions may remain stable in size; however, spontaneous inflammation and rupture may present with notable involvement of the surrounding tissue. Further, they may become infected by bacteria in the normal skin flora. Infected, fluctuant cysts are typically larger, erythematous, and more tender than sterile, inflamed cysts. The diagnosis of an epidermoid cyst is based on the clinical appearance of a discrete cyst or nodule, often with a central punctum, that is freely movable on palpation. All sebaceous cysts have walls consisting of stratified squamous epithelium similar to hair follicles. The distinction of epidermoid cysts is made histologically due to the prominent granular layer [3]. 

Among all skin cysts, pilar or trichilemmal cysts, a subtype of sebaceous cysts, are common dermal cysts but occur in less than 10% of the population, primarily affecting the skin of the scalp. Trichilemmal cysts never give rise to malignant lesions. Young individuals are more prone to developing trichilemmal cysts [1]. While pilar cysts have no known racial predilection, they are more often present in women than men [1]. The presentation is sporadic or inherited as an autosomal dominant trait. Patients with a genetic predisposition to pilar cysts are often younger and often present with multiple lesions simultaneously [1]. As to the patient's recollection in this study, the first cyst appeared on her ears as a child, which was surgically removed when she was in her 20s, so there is the possibility of familial origin. Congenital pilar cysts are associated with mutations in the phospholipase C delta one gene (PLCD1) [4]. Unfortunately, the patient did not partake in genetic studies to determine the genetic origins definitively. 

Pilar cysts are firm, slow-growing subcutaneous nodules that take several years to develop. They are most common on the head, especially the scalp. Pilar cysts are intradermal cysts derived from the root sheath of the hair follicle, located in the epithelium between the sebaceous gland and the arrector pili muscle. They are lined by stratified squamous epithelium lacking a granular cell layer and filled with keratin and breakdown products. Complications include proliferating trichilemmal cysts, the tumor form of pilar cysts, which results from less than 3% of all cases of pilar cysts. These lesions may ulcerate and become locally aggressive [1]. The patient, in our case, first noticed her present cysts in 1996. However, she denied pain and acknowledged that the lesions were uncomfortable, and noticed that they were slowly growing. In addition, she had a brother with a single cyst. She had one of the cysts ruptured in the past, which led to significant bleeding, and she subsequently went to the ER for treatment. 

The treatment of pilar cysts involves surgical excision of the cysts and the wall, which produces maximum benefit, leading to a decreased probability of recurrence. Inflamed, ruptured cysts that are not infected may resolve spontaneously without therapy, although they tend to recur. It is typically easier to remove pilar cysts than epidermoid cysts because the firm cyst wall does not easily rupture. Enucleated cysts generally appear as firm, smooth, white nodules. The diagnosis is confirmed through histology, as in this case. 

Excision is ideal without inflammation because inflammation is related to the friability of the cyst wall, which increases the chances of recurrence [3]. Therefore, it is advisable to wait until the inflammation has resolved before attempting excision. Triamcinolone acetonide injections can reduce inflammation in these lesions if required, with size reduction added benefit. In the past, this patient had a ruptured cyst complicated by bleeding, requiring emergent surgery. The history of ruptured cysts, irritations associated with the cysts, and cosmetic reasons warranted surgery for the patient in this study. 

This case is significant because eleven cysts ranging from 0.5 to 3.8 cm were removed from the scalp and jaw and histology confirmed that ten of them were pilar cysts while one was an epidermal inclusion cyst.

## Conclusions

Cystic lesions, regardless of etiology, call for medical intervention, whether for cosmetic reasons or for evaluation of a neoplastic process. Genetic testing for PLCD1 and P53 was not done to confirm the genetic origin but it is suspected that the pilar cysts observed in our patient are possibly hereditary due to a family member having a history of a similar cyst.

The patient underwent two separate procedures one month apart, with ten pilar cysts excised. The first surgery could not adequately remove all of the presenting cysts because of their location and the risk of contaminating the sterile field. During the first surgery, the patient was in a supine position with the removal of six total cysts. The patient was placed in the prone position for the second surgery, with the removal of five other cysts. Histology confirmed that ten were pilar cysts, and one was an epidermoid cyst.

Whether or not these excisions will be curative for this patient remains to be seen. However, as the cysts were removed before rupture, the likelihood of recurrence is comparatively low.
